# Efficacy of Iron-Rich Snacks in Improving Iron Status Among Adolescent Girls (10‒19 Years): A Systematic Review

**DOI:** 10.1016/j.advnut.2025.100549

**Published:** 2025-10-29

**Authors:** Hope Masanja, Haikael D Martin, Theresia Jumbe, Wanjiku N Gichohi-Wainaina

**Affiliations:** 1 Department of Food Biotechnology and Nutritional Sciences, Nelson Mandela African Institution of Science and Technology, Arusha, Tanzania; 2 Department of Human Nutrition and Consumer Sciences, Sokoine University of Agriculture, Morogoro, Tanzania; 3 WorldFish, Penang, Malaysia

**Keywords:** snack, iron status, hemoglobin, adolescent, girls

## Abstract

Iron deficiency anaemia (IDA) is the most common form of anaemia and the leading cause of years lived with disabilities (YLD) and disability-adjusted life years (DALYs) among adolescents. Recently, various iron-rich snacks have been developed as interventions to improve iron status among adolescents. This review aimed to evaluate the efficacy of natural, non-commercially fortified iron-rich snacks in improving iron status among adolescent girls aged 10–19 years. A systematic review of randomized controlled trials and quasi-experiments was conducted, focusing on iron status indicators including hemoglobin (Hb), serum ferritin (SF), transferrin saturation (TSAT), and soluble transferrin receptors (sTfR). Searches were performed in PubMed, ScienceDirect, EBSCO, Research4Life, and Google Scholar. Risk of bias was assessed using Cochrane tools, and study quality was evaluated with GRADE. Ten studies (five RCTs and five quasi-experiments) involving 24 to 211 participants were included. Nine studies had moderate risk of bias, mainly due to performance, detection, and reporting issues; one had high selection bias. Iron content of snacks varied, with nine studies out of ten reported Hb increases ranging from 0.45 to 2.28 g/dL. Only one study reported improvements in serum iron (from 25.482 ± 0.036 g/dL to 41.511 ± 0.033 g/dL) and ferritin (from 10.827 ± 0.192 ng/mL to 14.016 ± 0.103 ng/mL). These results indicate the potential of locally developed snacks to improve iron and Hb levels in adolescents. This review synthesizes evidence on food-based interventions, focusing on natural, non-fortified iron-rich snacks for adolescent girls. The findings demonstrate promising potential for these snacks to improve iron status and haemoglobin concentrations, underscoring their value as culturally acceptable, cost-effective, and sustainable complement to existing nutrition strategies. Although results are promising, more rigorously designed trials with comprehensive iron biomarkers are needed to confirm efficacy and support integration into adolescent nutrition programs.

## Graphical abstract

**Figure:**
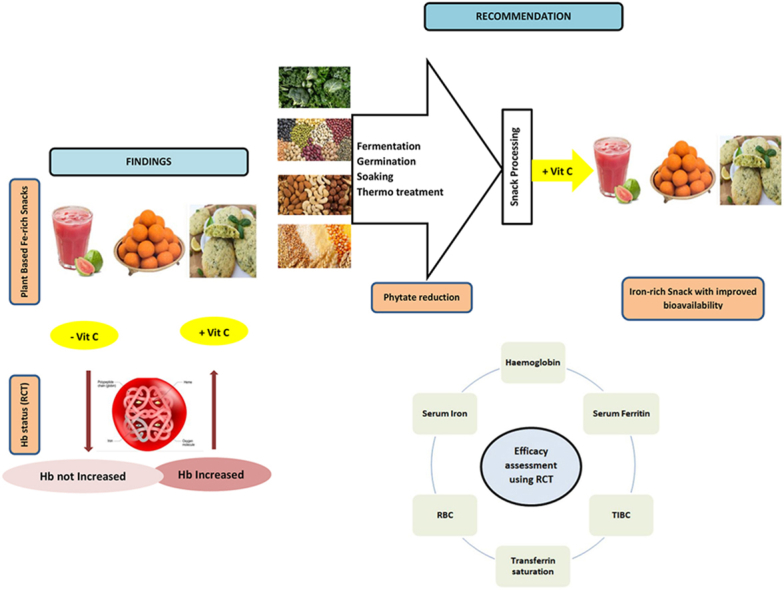

**Statements of significance**

This review focuses on the efficacy of iron-rich snacks made entirely from natural ingredients, providing context-specific evidence relevant to food-based strategies in low-resource settings, unlike prior reviews that emphasize commercially fortified products or supplementation.

## Introduction

Globally, ∼30% of females aged 15–49, including late adolescent girls, are affected by anemia [1], with iron deficiency (ID) being the leading cause [2]. Anemia during adolescence can lead to developmental delays, poor school performance, reduced attention to tasks [3], and long-term impacts on productivity and overall quality of life. Moreover, iron deficiency anemia (IDA) is the leading cause of years lived with disabilities and disability-adjusted life years among adolescents [4,5]. Inadequate iron intake among adolescents has been widely reported [6], making increased iron consumption a key strategy to improve iron status and prevent IDA. Due to the complexity of ID, multiple interventions, such as food fortification, dietary diversification, iron supplementation, and nutrition education, are recommended [7].

School feeding programs are also increasingly recognized as an effective platform for delivering essential nutrients to school-aged children [8,9]. Most adolescents spend the greater part of their day in school, making the school food environment a critical source of both energy and nutrients. However, in many contexts, school meals fall short of dietary recommendations, often prioritizing inexpensive calorie provision over dietary diversity and nutrient quality [[10], [11], [12], [13], [14], [15], [16], [17], [18], [19], [20]].

Leveraging adolescents’ common snacking habits, iron-rich snacks offer a practical approach to improving nutrition in this age group [[16], [17], [18]]. Although systematic reviews have examined the effects of supplementation and commercially fortified foods on ID and IDA among adolescent girls [[19], [20], [21]], evidence on food-to-food fortified snacks remains limited. In this context, the present systematic review aims to synthesize available evidence on the efficacy of noncommercially fortified iron-rich snacks as a strategy to enhance iron status among adolescent girls.

## Methods

### Design

This systematic review was conducted following the PRISMA guidelines [21]. Articles of interest were identified by 2 independent reviewers who conducted a comprehensive search of PubMed, ScienceDirect, Research4Life, and EBSCO databases, with no time restrictions. To ensure thorough coverage, a gray literature search was also performed using Google Scholar. The article searches were carried out in July 2023. On 30 September, 2024, the same search was repeated to capture any relevant studies published since July 2023. Additionally, the reference lists of included studies were examined to identify any further relevant articles that could supplement the structured and less structured electronic database searches. In cases of disagreement or discrepancies, the third and fourth reviewers were consulted to reach a consensus.

### Eligibility criteria

In this systematic review, the criteria utilized for defining the population, intervention/exposure, comparator, outcome, and types of studies, as well as study design, for inclusion are outlined in Table 1 [22]. Additionally, the inclusion criteria involved full-text studies published in peer-reviewed journals, proceedings, and reports from authoritative organizations and agencies, all available in English. We included only studies that enrolled apparently healthy participants, with no health conditions other than anemia. Studies involving animals or carried out in a mixed population (e.g., female and male), if not presenting results separately for sub-groups, were excluded. After screening for eligibility, all relevant information and data were extracted from the remaining studies.

**TABLE 1:** The criteria applied for a population, intervention/exposure, comparator, outcome, and types of studies

		Inclusion criteria	Exclusion criteria
(P) Population	Adolescent girls	Apparently healthy adolescent girls who are aged 10‒19 y; healthy, nonpregnant; nonbreastfeeding	Subjects with age below or above 10‒19 y and any known health condition other than dietary anemia, including those that may influence iron status, such as sickle cell anemia, hemoglobinopathies, and others
(I) Intervention^1^	Iron-rich snack	An iron-rich, food-based snack designed specifically to improve iron status, made entirely from natural food ingredients and available in various forms, including solid, liquid, and semisolid. This snack should not be commercially fortified but instead relies on the inherent nutritional content of its ingredients to boost iron concentrations.	Commercially fortified snack; produced for purposes other than improving iron status; produced from nonfood ingredients
(C) Comparison	Efficacy of iron-rich snack(s) against other noniron-rich snack(s) or other approaches to address iron deficiency (ID), including iron supplementation, normal diet, nutrition education, or placebo	Efficacy of iron-rich snack(s) compared to noniron-rich snack(s) or other approaches to address ID, including iron supplementation, normal diet, or placebo	Efficacy that is attributed to other interventions, i.e., population receiving iron-rich snacks and other intervention(s) at the same time
(O) Outcome	Iron status (concentration of serum ferritin, transferrin saturation), hemoglobin concentration, hematocrit, anemia, ID, and ID anemia.	Outcome measures assessed before and after intervention	Outcome measures were only assessed at a single time point; outcome measures were presented for a mixed population.
(T) Type of Study	Quasi-experiments and randomized controlled trials	Experimental method with pre- and posttesting of this review outcome. Including both randomized and nonrandomized assignment of study participants into intervention group(s) and comparison group(s).	Studies with no pre- and posttest of the study outcome.

1 Snack refers to food or caloric beverages between regular meals or energy-dense, nutrient-poor foods high in sodium, sugar, and/or fat, such as cookies, cakes, sugar-sweetened beverages, and chips [22].

### Search strategy

**FIGURE 1:**
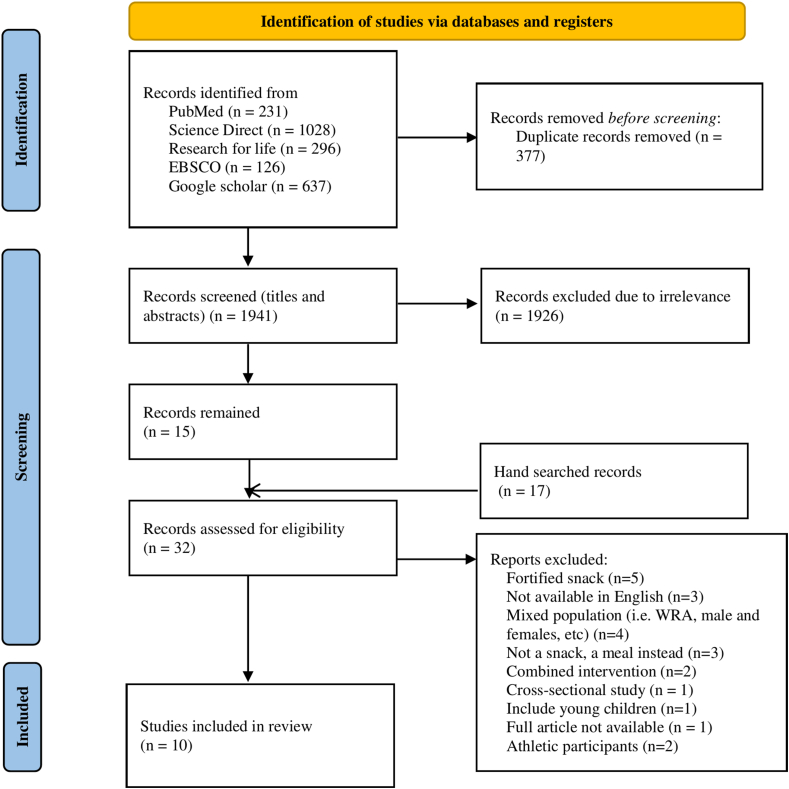
Flow chart of studies identification.

The search string was developed using Dundee search generator, https://libguides.dundee.ac.uk/c.php?g=233786&p=4760265, by means of the keywords and their corresponding synonyms: snacks, snack bar, women, female, girl, adolescent, iron, iron status, heme, ferritin, transferrin, and anemia, Table 2. The procedure of study identification in selected databases is presented in Figure 1. Titles from all databases were downloaded, and duplicates were removed. Based on the inclusion and exclusion criteria, studies were selected by 2 independent reviewers (HM and WNG-W) through the screening process of titles, abstracts, and full text. In case of disagreement and/or discrepancies, the third and fourth reviewers were involved (HDM and TJ).

**TABLE 2:** Detailed searching strategy for each electronic database used

Serial Number	Source	Search strategy
1	PubMed	(“snack∗” OR “snack bar”) AND [(women OR female∗ OR girl∗ OR adolescent∗) AND (“iron” OR “iron status∗” OR heme∗ OR haem∗ OR ferritin OR transferrin OR anaemia OR anemia)]
2	Science Direct:	(snack! OR “snack bar!”) AND (adolescent!) AND (“iron!” OR “iron status!” OR heme! OR haem! OR ferritin OR anemia)
4	R4L(AGORA)	(“snack∗” OR “snack bar”) AND {∖[∖(women OR female∗ OR girl∗ OR adolescent∗∖) AND ∖(“iron” OR "iron status∗” OR heme∗ OR haem∗ OR ferritin OR transferrin OR anaemia OR anemia∖)∖]}
5	EBSCO	(“snack∗” OR “snack bar”) AND [(women OR female∗ OR girl∗ OR adolescent∗) AND (“iron” OR “iron status∗” OR heme∗ OR haem∗ OR ferritin OR transferrin OR anaemia OR anemia)]
6	Google scholar	(snack OR “snack bar”) AND [(women OR female OR girl OR adolescent) AND (“iron” OR “iron status” OR heme OR haem OR ferritin OR transferrin OR anaemia OR anemia)] sort by relevance

Abbreviation: R4L(AGORA), Research4Life (Access to Global Online Research on Agriculture.

### Data extraction

A data extraction form in Microsoft Excel, adopted from the Cochrane Collaboration, was used by the review team. The extraction process was conducted by 1 author (HM) and then repeated by 2 other authors for 30% of the papers included in the study. The data extracted were coded accordingly before proceeding with data analysis. Data extraction included the following key variables:

- The publication details (author, date of publication, study design, study period, country, and sample size)
- Study population and demographics, such as age, settings (for example, secondary school, slums, and university)
- Iron-rich snack products used as an intervention, including food or caloric beverages between regular meals or energy-dense, nutrient-poor foods high in sodium, sugar, and/or fat, such as cookies, cakes, sugar-sweetened beverages, and chips [22]
- Details about the control group and other group(s) (where present), such as receiving the placebo or normal diet, nutrition education, iron supplementation, etc.
- Concentration of iron (milligrams per day) on formulated iron-rich snack product (iron, both heme and nonheme if described)
- Intervention duration in days
- Time points for outcome measurement
- Hemoglobin (Hb) concentration in grams per deciliter and/or other indicators for assessing iron status (serum ferritin, transferrin saturation, hematocrit, anemia, ID, and IDA) measured at different time points and significance change (*P* value if reported)

Whenever information was not available, insufficient, or unclear within the full text of the article, the corresponding author of the article was contacted to obtain the necessary information.

The quality of the included articles was assessed through an approach adopted by Cochrane, Grading of Recommendations, Assessment, Development and Evaluation (GRADE) approach [23]. GRADE focuses on assessing the certainty (or quality) of a body of evidence. The assessment was done through 5 domains: risk of bias, inconsistency, indirectness, imprecision, and publication bias. It was conducted through the GRADE Working Group’s software, GRADEpro Guideline Development Tool (GDT) (www.gradepro.org). The risk of bias assessment was conducted using the revised Cochrane risk-of-bias tool for randomized trials (RoB 2.0) [24] and the Cochrane risk of bias assessment tool for nonrandomized studies of interventions [25]. Risk of bias for each study was assessed within 5 domains: bias due to deviations from intended interventions, bias arising from the randomization process, bias in the measurement of the outcome, bias due to missing outcome data, and bias in the selection of the reported result. Trials were included in the study regardless of the risk of bias. Two reviewers (HM and WNG-W) assessed the quality of the studies included in the review, including the risk of bias; in case of disagreement, the third and fourth reviewers were involved (HDM and TJ). Mean difference was used as the effect measure for each study outcome.

## Results

### General study description

Table 3 [[26], [27], [28], [29], [30], [31], [32], [33], [34], [35]] presents general characteristics of studies included in this review: 5 randomized controlled trials (RCTs) and 5 quasi-experiments. Nine studies out of 10 studies included in this review were from developing countries in Asia: 5 from Indonesia, 2 from India, 1 from Bangladesh, and 1 from Pakistan. No study area was mentioned in 1 quasi-experimental study [35]. Of the studies that mentioned the period when they conducted the studies, the most recent was from October to November 2021, and the oldest was conducted in January 2013.

**TABLE 3:** The general characteristics of the studies included in the systematic review

Authors and year	Sample size	Participants characteristics	Study design	Studied intervention	Intervention duration (days)	Groups	Outcome measure	Pre-intervention (mean)	Postintervention (mean)	Key findings
Mega et al. [26], 2019	32	Secondary school adolescent girls, 15‒18 y	RCT	Red guava juice vs. ferrous sulfate	14	1. Red guava group 2. Iron supplementation group	Hemoglobin (Hb) (g/dL); hematocrit (%)	1. 11.4; 35 2. 11.3; 34.2	1. 12.3; 39 2. 12.3; 37.8	Hb and Hct concentration significantly increased among anemic adolescent females consuming red guava juice, by 0.9 g/dL and 4% respectively (*P* < 0.005). Iron supplementation produced comparable improvements in Hb and Hct concentrations as red guava juice, increasing by 1.0 g/dL and 3.6%, respectively (*P* = 0.1).
Ardela et al. [27], 2023	24	Secondary boarding schoolgirls, 15‒17 y	RCT	Mixture of 100 mL of water with 100 g fresh guava (red guava juice 1) vs. mixture of 100 mL of water with 150 g fresh guava (red guava juice 2) vs. mixture of 100 mL of water with 200 g fresh guava (red guava juice 3) vs. no intervention (control)	5	1. KP1 2. KP2 3. KP3 4. KP0	Hb (g/dL)	1. 12.9 2. 12.4 3. 12.1 4. 13.0	1. 12.5 2. 13.1 3. 12.6 4. 12.0	Red guava juice at a dose of 250 mL increases blood Hb concentration during menstruation, by 0.45 g/dL (*P* = 0.000), and no significant difference in efficient on increasing Hb concentration when compared to doses beyond this (*P* = 0.561).
Singh et al. [28], 2014	30	Adolescent girls of university undergraduate classes, 16‒19 y	RCT	iron-rich laddu vs. IFA vs. no intervention (control)	45	1. Group A 2. Group B 3. Group C	Hb concentration (g/dL)	1. 9.8 2. 9.7 3. 9.4	1. 12.1 2. 11.9 3. 9.9	Laddu improved adolescent girls’ Hb concentration, from 9.81 g/dL to 12.05 g/dL (*P* < 0.001). No difference between intervention groups (laddu vs. IFA) on mean Hb gain (*P* > 0.05)
Patil et al. [29], 2014^1^	52	Adolescent girls, regardless of whether they attend school or not, residing in the slum, adopted by the urban health training center area, 11‒18 y	RCT	Nutrition education vs. ferrous sulfate (with 100 mg elemental iron) and folic acid tablets (0.5 mg) vs. iron-rich laddu (with 14 mg iron per serving)	30	1. Group 1 (nutritional education) 2. Group 2 (ferrous sulfate and folic acid tablets) 3. Group 3 (iron-rich food in the form of laddus)	Hb concentration (g/dL)	1. 10.5 2. 10.7 3. 10.4	1. 10.4 2. 11.3 3. 10.4	Adolescent girls who consumed laddu did not show any rise in the Hb concentration, maintained 10.4 g/dL, which was associated with less bioavailability of iron in the laddu. Iron supplementation showed a significant rise in the mean Hb concentration compared to other interventions (*P* < 0.001)
Akbar et al. [30], 2024	204	Adolescent girls from several schools and colleges within the Sargodha region, 13‒19 y	RCT	Sweet basil leaf (SBLP) powder-enriched cookies (B3) vs. ferrous sulfate (B1) vs. placebo (control) (B0)	120	1. SBLP powder-enriched Cookies (B3) 2. Ferrous sulfate (B1) 3. Placebo (control) (B0)	Serum iron (g/dL), Serum ferritin (ng/mL), transferrin saturation (TSAT) (%), total iron-binding capacity (TIBC) (*μ*g/dL), red blood cells (M/*μ*L), hemoglobin (Hb) (g/dL), Hematocrit (Hct) (%), MCV (fL), MCH (pg), MCHC (g/dL)	1. 25.5, 10.8, 11.0, 390.7, 4.1, 11.4, 31.4, 77.0, 27.2, 33.5 2. 25.5, 10.8, 11.0, 390.7, 4.1, 11.4, 31.4, 77.0, 27.2, 33.5 3. 25.5, 11.6, 11.0, 391.2, 4.1, 11.4, 31.3, 77.0, 27.2, 33.4	1. 41.5, 14.0, 13.0, 374.8, 4.3, 12.7, 33.6, 78.4, 28.7, 35.5 2. 39.9, 13.6, 12.7, 376.6, 4.3, 12.5, 33.3, 78.2, 28.7, 35.3 3. 24.8, 11.1, 10.8, 398.3, 4.1, 10.9, 31.1, 76.7, 27.0, 31.6	The highest increase in serum ferritin (3.19 ng/mL), serum iron (16.03 g/dL), Hb (1.31 g/dL), and TSAT (2.01%), along with a decrease in TIBC (15.87 μg/dL), was observed in a group that received 16 g SBLP compared to other groups (*P* < 0.05). The vice versa was observed in the control group.
Pibriyanti et al. [31], 2021	30	Adolescent girls in Islamic boarding school, 15‒19 y	Quasi-experiment	Beetroot juice	7	1. 60 g of beetroot is consumed in the form of 200 mL/d	Hb concentration (g/dL)	12.0	13.6	Consumption of beetroot juice increased Hb concentration by 1.57 g/dL (*P* = 0.001).
Maharani et al. [32], 2022	21	Seventh-grade students, teenagers	Experiment with a pre-test and posttest design	Green bean juice vs. red guava pudding and green bean juice vs. gelatin (control)	7	1. Green bean juice as much as 300 mL 2. Combination of guava pudding and green bean juice of as much as 300 g 3. Gelatin as much as 50 g	Hb concentration (g/dL)	1. 11.1 2. 11.0 3. 11.1	1. 13.8 2. 14.6 3. 12.1	Both treatments increased Hb concentrations (*P* value 0.000). The effect of the combination of red guava pudding and green beans on increasing Hb concentrations was higher than that of green bean juice only, by 3.6 g/dL and 2.7 g/dL, respectively (*P* = 0.000).
Khanam et al. [33], 2022	211	High school early adolescent girls, 12‒14 y	Quasi-experimental design	Moringa pakora (oil-fried snack) vs. calorie-matched meal without moringa pakora (control)	6 mo	1. Moringa pakora (oil-fried snack, 150 g). Served with 30 g of rice and 25 g of concentrated dal, as well as nutrition education 2. 30 g rice, 25 g concentrated, dal and 25 g fried potato (aluvaji) as well as nutrition education	Hb concentration (g/dL)	1. 12.0 2. 11.8	1. 13.3 2. 12.6	Moringa leaves, in addition to the diet, increased the Hb concentration, from 12.04 to 13.31 g/dL, among adolescent girls positive change in the Hb concentration (intervention compared to control): DiD coef = 0.41; 95% CI: 0.14, 0.76; *P* = 0.009)
Ariendha et al. [34], 2022	64	Female adolescents at STIKES Yarsi Mataram, age not mentioned	One group pre-post-test experimental research design	Moringa leaf cilok vs. blood-boosting tablets (control)	15	1. Moringa leaf cilok; 2. Blood-boosting tablets (control)	Hb concentration (g/dL)	1. 11.5 (10.1–11.8); median (min - max) 2. 11.0 (10–11.9); median (min - max)	1. 12.5 (12.1–13.0); median (min - max) 2. 13.6 (11–17.0); median (min - max)	Moringa leaf cilok increased Hb concentration among adolescent girls by 1.0 g/dL (*P* ≤ 0.000). Blood-added tablets increased Hb concentration by 2.6 g/dL (*P* = 0.001), 2 times more than moringa leaf cilok.
Tirtawati et al. [35], 2021	100	Adolescent females, age not mentioned	Pre-post-test 1-group design	Moringa oleifera teabags	30	1. Moringa tea bag dipped in 250 mL of hot water, and then added 2 teaspoons of granulated sugar	Hb concentration (g/dL)	10.7	11.6	Moringa leaf tea bags increased Hb concentration in adolescent females by 0.92 g/dL (*P* < 0.05)

Abbreviations: CI, confidence interval; IFA, iron-folic acid; min, max, minimum, maximum; RCT, randomized controlled trial; TSAT, transferrin saturation; B0, Control; B1, Ferrous sulfate; B3, SBLP powder-enriched Cookies; DiD coef, Difference in Difference Coefficient; KP1, Mixture of 100 mL of water with 100 g fresh guava (red guava juice 1); KP2, Mixture of 100 mL of water with 150 g fresh guava (red guava juice 2); KP3, Mixture of 100 mL of water with 200 g fresh guava (red guava juice 3); KP0, No intervention (control); MCH, Mean Corpuscular Hemoglobin; MCHC, Mean Corpuscular Hemoglobin Concentration; MCV, Mean Corpuscular Volume.

1 Randomization status was provided on request.

### Study interventions and comparisons

#### RCTs

The intervention products in this review were red guava juice, laddu, and sweet basil leaf powder (SBLP)-enriched cookies. Laddu (sometimes called ladoo) is a traditional Indian confectionery made from a mixture of ingredients formed into round balls. It is defined by its shape rather than its ingredients, which can vary widely. The ingredients are typically held together by sugar syrup or jaggery and may be fried [36]. The comparison groups received iron supplements, nutrition education, placebos, or no intervention.

Two RCT studies [2] used red guava juice as an intervention. One, provided adolescent girls with 100 mL of red guava juice containing ∼0.69 mg iron, provided once daily for 14 d, secondary school adolescent girls [26]. The intervention group was compared against a group that received an iron supplement in the form of ferrous sulfate containing 60 mg elemental iron, daily for 14 d. The other RCT, a different volume of red guava juice made by mixing 100 mL of water with 100 g, 150 g, and 200 g fresh guava was given to secondary boarding school adolescent girls once daily for 5 d [27]. The comparator was a control group that continued consuming their usual meals.

The other 2 RCTs employed laddu as the snack vehicle for iron intervention [28,29]. In detail, a 100 g iron-rich laddu containing 15.56 mg iron, prepared from pearl millet flour (20 g), lotus stems flour (10 g), soybean flour (10 g), niger seeds (10 g), jaggery (50 g), fat (5 g) and water (15 mL) was served along with 200 mL lemon water once daily for 45 d [28]. This trial used 2 groups as comparison groups. One comparison group received iron-folic acid tablets containing 60 mg elemental iron daily for 45 d, whereas the other group received no intervention and served as a control. The other RCT, conducted by Patil et al. [29] (2018), provided participants with 2 60 g laddus per day, each containing 14 mg iron, amounting to a total daily intake of 28 mg over 30 d. Laddu from the second RCT were prepared from garden cress seeds (locally called “haliva”) 250 g, dry coconut 500 g, and jaggery 750 g, which contained a total of 349 mg iron. The trial had 2 comparison groups. One group was provided with ferrous sulfate and folic acid tablets containing 100 mg elemental iron and 0.5 mg folic acid daily for 30 d. The second group received education on the role of nutrition in overcoming anemia, delivered through printed materials, lectures, and discussions, once a week for over 30 d.

The remaining RCT used SBLP-enriched cookies with 16 g of SBLP/100 g cookie as an intervention providing 7 mg iron. The cookies were provided with orange/ strawberry juice to study participants for 120 d. For this fifth RCT, 2 comparison groups were established: 1 group received ferrous sulfate (12.7 FeSO4·7H2O/100 g), whereas the second group served as the control (placebo). Both groups were compared against the intervention group that received SBLP cookies.

#### Quasi-experiments

Interventions included under the quasi-experiments were beetroot juice, green bean juice, moringa pakora (oil-fried snack), moringa leaf cilok, and moringa oleifera teabags. Pakora, also known as pakodas or bhajji, are deep-fried fritters that are a popular snack and street food widely consumed across India and parts of Southeast Asia [36]. Cilok, an Indonesian snack, is made by blending all-purpose flour and tapioca flour with other selected ingredients. The mixture is shaped into balls and cooked either by boiling or deep frying [36].

Two of the quasi-experimental studies employed a pre-post (within-group) design, wherein the efficacy of the intervention was evaluated by comparing outcome measures obtained before and after the intervention within the same group. One trial had the beetroot juice as an intervention, containing 60 g of beetroot, consumed in the form of 200 mL of juice, daily for 7 days [31]. The other trial was conducted by Tirtawati et al. [35] (2021), where participants consumed 2 sachets of moringa teabags (3 g each) daily, 1 in the morning and 1 in the afternoon. Each sachet was steeped in a cup of hot water (250 mL) and sweetened with 2 teaspoons of sugar (10 g), consumed over a 30 days period.

Between the quasi-experimental studies with comparison groups, 1 evaluated the effects of green bean juice (300 mL daily for 7 d) against 2 comparators [32]: a combination of guava pudding and green bean juice (300 g) and a gelatin supplement (50 g) as the control. Another study assessed moringa pakora, an oil-fried snack prepared from 150 g of fresh moringa leaf granules mixed with chili, lentils, onion, and salt [33]. Each portion contained 3 pakoras (50 g each), providing ∼282 kcal/d, served together with 30 g rice and 25 g dal, alongside nutrition education. The comparison group received a similar meal consisting of 30 g rice, 25 g dal, and 25 g fried potato (aluvaji) with nutrition education. A third quasi-experimental study tested moringa leaf cilok, a snack prepared from 4 g moringa leaf flour, 50 g flour, 50 g tapioca flour, 2 g salt, 70 mL water, 2 g mushroom broth, and leek [34]. Participants consumed the snack 5 d a week for 30 d, and outcomes were compared against a control group that received blood-boosting tablets.

### Efficacy of interventions

Table 3 also presents findings and conclusions of each RCT and quasi-experiment included in this systematic review. All interventions tested focused on Hb concentration as an outcome indicator among adolescent girls, except for 2 studies by Akbar et al. [30] (2024) and Mega et al. [26] (2019). Generally, 1 RCT by Patil et al. [29] (2014) among the shortlisted studies on the efficacy of an iron-rich snack (laddu) was not found to be efficacious in increasing the Hb concentration among study participants, with no significant change in Hb concentration. The conclusion drawn was that the lack of efficacy in raising Hb concentration may be attributed to the lower bioavailability of iron in the food vehicle-laddu. On the contrary, the remaining 9 studies reported significant improvements in Hb concentrations between intervention groups, with mean increments ranging from 0.45 g/dL to 2.28 g/dL, thereby demonstrating the efficacy of the interventions. Additional benefits were also observed in other hematological outcomes in 1 RCT. In this study, participants receiving SBLP-enriched cookies showed the greatest increase in serum iron, serum ferritin, and transferrin saturation, along with a decrease in Total iron-binding capacity (TIBC) (*P* < 0.05) [30].

Similar to the RCT, all interventions under quasi-experiment studies were reported as efficacious in improving Hb concentration among adolescent girls, Table 3.

### Quality appraisal

Low quality was observed in RCT studies, which were mostly due to high risk of bias and imprecision (Supplementary Table 1). Unclear information on blinding of participants or outcomes assessment status, as well as randomization status in 1 study, mostly contributed to the risk of bias. Imprecision was due to a small number of study participants and a lack of enough information concerning the compliance with the intervention. Nonrandomized studies included had a high risk of bias and imprecision due to similar reasons as RCT studies. Further, all studies also had inconsistency problems due to differences in study settings.

### Risk of bias

The results of the risk of bias assessment are outlined in Table 4 [[26], [27], [28], [29], [30], [31], [32], [33], [34], [35]]. Nine [[26], [27], [28],^30^] out of 10 had moderate risk, mainly due to performance, detection bias, and reporting bias. Only 1 study had high bias risk, which was due to selection bias [29]. Due to the high risk of selection bias, communication was done with the corresponding author to confirm the randomization status of the study participants into groups. No attrition bias was observed in all RCTs, and no selection bias was anticipated except for the RCT conducted by Patil et al. [29] (2014).

**TABLE 4:**
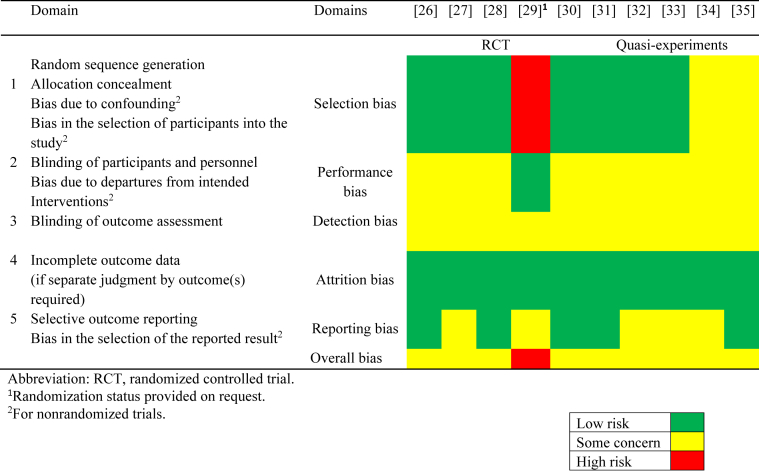
Risk of bias assessment for the randomized controlled trials included in the systematic review, conducted by using the Cochrane risk-of-bias tool for randomized trials

## Discussion

This systematic review aimed to evaluate the efficacy of noncommercially fortified iron-rich snacks as an intervention to improve iron status among adolescent girls. In summary, evidence from studies included in this review demonstrates that locally developed iron-rich snacks have potential in improving iron status markers, including Hb concentrations among adolescent girls, even during menstruation. Iron bioavailability was identified as a crucial factor influencing the efficacy of the plant-based iron-rich snack. In the next section, we discuss the key issues related to these alternative snacks that have emerged from this review.

### Bioavailability

Ensuring high iron bioavailability is critical for the efficacy of iron-rich snacks in improving iron status. Both plant food sources and animal food sources can be used to develop iron-rich snacks. Animal source snacks usually provide higher concentrations of bioavailable iron, though they are likely to be costly, particularly among low-income consumers [37]. In contrast, the iron bioavailability of plant-based snacks is limited due to the presence of potent iron absorption inhibitors, such as phytate, polyphenols, tannins, and oxalic acid [38]. To improve iron bioavailability in plant-based snacks, incorporation of iron absorption enhancers (vitamin C or animal tissues), use of traditional food processing methods such as fermentation, malting, soaking, thermo processing, and the addition of enzymes such as phytase have also been proven effective [[39], [40], [41], [42], [43]]. These methods reduce inhibitors and enhance iron absorption, although some nutrient loss may occur, so selecting optimal processing methods that balance these considerations is important. For example, although vitamin C is a potent enhancer of nonheme iron absorption, it is highly thermolabile, leading to losses in content, bioactivity, and nutritional quality during processing and storage [39]. Hence, it is crucial to use processing methods that ensure high vitamin C retention [44,45]. On the contrary, traditional food processing methods require further investigation to assess optimal time, temperature, and humidity combinations. Moreover, incorporation of vitamin A may improve iron status by regulating iron metabolism, supporting the overall efficacy of iron interventions [6,46]. Both methods improve the efficacy of plant-based iron-rich snacks, as shown by 1 RCT included in this review [29].

### Fortification and supplementation

Fortification of foods has been proven to be efficacious in reducing anemia, IDA, and ID [47]. Incorporating iron into commonly consumed foods is a practical and effective strategy to improve population iron status, especially in groups at high risk of deficiency. However, developing iron-fortified snacks requires advanced technology to avoid taste and other sensory qualities perturbations. Incorporating enzymes such as phytase into plant-based foods can enhance iron absorption and has been shown to generate cost savings of ∼10–30% [48]. However, the additional expense of phytase fortification may pose challenges to affordability, particularly in low- and middle-income settings. Besides fortification, routine iron supplementation is additionally practiced in some countries for adolescent girls, but commercial iron supplements have potential side effects due to free iron radicals affecting the gut lumen and mucosal area of the intestine [38]. Taken together, these findings highlight that although iron fortification, supplementation, and the use of enhancers such as phytase offer promising avenues to reduce anemia and ID, their success depends on striking a balance between efficacy, affordability, and acceptability. Moving forward, innovations in product formulation, coupled with strategies to reduce costs and maintain sensory quality, are critical. Equally important will be investments in public awareness and behavior change to ensure uptake and sustained impact.

### Nutritional balance and risks

Although micronutrient-rich snacks, including those fortified with iron, have the potential to strengthen school food systems by providing nutritious, safe, and affordable diets, they also carry the risk of increasing calorie intake disproportionately to improvements in micronutrient status [16,18,48,49]. Designing snacks that are both iron-rich and balanced in macronutrients is challenging, yet it is crucial to consider the overall nutritional profile of the proposed snack, as excessive consumption of high-calorie products may contribute to an increased risk of overweight [50,51]. Snack formulations should also balance iron needs with the tolerable upper intake level (45 mg/d for individuals aged 19 and above) to avoid toxicity [52]. Despite the resource-intensive nature of RCTs, conducting them remains essential to evaluate the efficacy and safety of such snacks.

### Snack acceptability and affordability considerations

For iron-rich snacks to be accepted, it is important to align them with context-specific preferences such as flavor, texture, packaging, and other attributes familiar to the target population [53]. Integrating these snacks into adolescents’ existing eating habits, particularly within school snacking routines, can improve uptake. For example, given adolescents’ snacking behaviors and the frequent availability of snack options in school environments, iron-rich snacks should be offered in a similar manner to other commonly consumed snacks.

Plant-based snacks are affordable, widely accessible, and sustainable, making them a promising option for scalable nutrition interventions to improve dietary iron intake and overall nutrition outcomes [54,55]. Although affordability can be addressed through the use of low-cost ingredients and appropriate processing methods [56,57], the challenges of sourcing quality inputs and ensuring product stability during storage and distribution remain significant[58]. Striking a balance between nutritional quality, product stability, and cultural acceptability is therefore critical for the success and sustainability of such interventions.

### Limitations

This review has several limitations. First, it included a limited number of RCTs, most of which were conducted in Asia, restricting the generalizability of the findings beyond Asia. RCTs, with their high internal validity and rigorous control, are the gold standard for assessing efficacy [59]. More RCTs across diverse geographies would have further strengthened the study’s conclusions. Second, Hb, often used as the primary outcome, is influenced by inflammation, infection, and other nutrient deficiencies, underscoring the importance of using more specific and sensitive iron biomarkers for accurate assessment [60]. Third, considerable heterogeneity existed across studies in terms of the food matrix of the iron-rich snacks, intervention duration, outcome measures, settings, and inclusion criteria, making comparisons difficult. Moreover, the studies differed in the food matrix of the iron-rich snacks used as interventions, which is important as it influences iron absorption and the overall efficacy of the snacks. Finally, none of the studies reported on antinutritional factors in the plant-based snacks, despite their potential impact on iron absorption and overall efficacy.

In conclusion, this review uniquely highlights the potential of natural, noncommercially fortified iron-rich snacks to improve Hb concentrations and overall iron status among adolescents. These findings underscore the translational potential of incorporating iron-rich snacks into adolescent nutrition interventions, including integration within school feeding programs and leveraging local food systems. Achieving effective and sustainable interventions will require multidisciplinary collaboration across nutrition, food science, public health, and policy sectors. Future research should prioritize scalable, affordable, and culturally acceptable interventions to maximize public health impact.

## Author contributions

The authors’ responsibilities were as follows – HM, TJ, WNG-W: designed research, developed the protocol, and registered the review on PROSPERO; HM, HDM, WNG-W: conducted research; HM: analyzed data, wrote the first draft, and had primary responsibility for final content; and all authors: read and approved the final manuscript.

## Data availability

All data included in this review were extracted from publicly available articles included in this review.

## Declaration of AI and AI-Assisted Technologies in the Writing Process

During the preparation of this work the author(s) used ChatGPT in order to enhance grammar and language. After using this tool/service, the author(s) reviewed and edited the content as needed and take(s) full responsibility for the content of the publication.

## Funding

The authors received the publication funds through contributions done by different funders to the CGIAR Trust Fund: https://www.cgiar.org/funders/ and the Better Diets and Nutrition Science Program: https://www.cgiar.org/cgiar-research-porfolio-2025-2030/better-diets-and-nutrition/.

## Conflict of interest

The authors report no conflicts of interest.
