# Supplementary material for: Efficacy of Iron-Rich Snacks in Improving Iron Status Among Adolescent Girls (10‒19 Years): A Systematic Review
Source: Adv Nutr. 2025 Oct 29;16(12):100549. doi: 10.1016/j.advnut.2025.100549 (PMC12677165; doi:10.1016/j.advnut.2025.100549)
Supplement: Multimedia component 1 [file mmc1.docx]

**Supplementary Table1: Assessment of the certainty of the body of evidence for outcomes included in this study**

| **Certainty assessment** | | | | | | | **№ of patients** | | **Effect** | | **Certainty** | **Importance** |
| --- | --- | --- | --- | --- | --- | --- | --- | --- | --- | --- | --- | --- |
| **№ of studies** | **Study design** | **Risk of bias** | **Inconsistency** | **Indirectness** | **Imprecision** | **Other considerations** | **iron rich snack** | **none** | **Relative (95% CI)** | **Absolute (95% CI)** |  |  |
| **Haemoglobin (follow-up: range 5 days to 120 days; assessed with: blood)** | | | | | | | | | | | | |
| 5 | randomised trials | serious^a^ | not serious^b^ | not serious | serious^c^ | none | 130 | 212 | - | mean **0.7 g/dl higher** (0.6 higher to 0.9 higher) | ⨁⨁◯◯ Low^a,b,c^ | CRITICAL  ^g^ |
| **Haemoglobin (follow-up: range 7 days to 180; assessed with: blood)** | | | | | | | | | | | | |
| 5 | non-randomised studies | serious^d^ | serious^b^ | not serious | serious^c^ | all plausible residual confounding would reduce the demonstrated effect dose response gradient | 289 | 137 | - | mean **1.8 higher** (1.7 higher to 1.9 higher) | ⨁⨁⨁◯ Moderate^b,c,d^ | CRITICAL  ^g^ |
| **Serum iron (follow-up: range 5 days to 120 days; assessed with: blood)** | | | | | | | | | | | | |
| 1 | randomised trials | not serious | not serious^b^ | not serious | serious^e^ | none | 68 | 136 | - | mean **16.03 g/dl higher** (0 to 0 ) | ⨁⨁⨁◯ Moderate^b,e^ | CRITICAL  ^g^ |
| **Serum ferritin (follow-up: range 5 days to 120 days; assessed with: blood)** | | | | | | | | | | | | |
| 1 | randomised trials | not serious | not serious^b^ | not serious | serious^e^ | none | 68 | 136 | - | mean **3.19 ng/mL higher** (0 to 0 ) | ⨁⨁⨁◯ Moderate^b,e^ | CRITICAL  ^g^ |
| **Hemotocrit (follow-up: range 5 days to 120 days; assessed with: blood)** | | | | | | | | | | | | |
| 2 | randomised trials | serious^a^ | not serious | not serious | serious^f^ | none | 84 | 152 | - | mean **3.1 % higher** (2.9 higher to 3.1 higher) | ⨁⨁◯◯ Low^a,f^ | IMPORTANT  ^h^ |

**CI:** confidence interval

#### Explanations

a. Serious risk of bias: Blinding of participants or outcomes assessment status was unclear contributing the most to the risk of bias

b. Serious inconsistency: Dietary differences among study participants, difference in iron content in snacks used as intervention, administration of the intervention, duration of intervention contributed to the inconsistency of change in outcomes at the end of studies

c. Serious imprecision: Most studies has few number of participants and actions to ensure compliance to the intervention was unclear

d. Most confounders for iron status were not taken cared in all studies

e. Serious imprecision: Actions to ensure compliance to the intervention was unclear

f. Serious imprecision: One study had low number of participants and actions to ensure compliance to the intervention was unclear in both studies
